# 2-Deprenyl-Rheediaxanthone B Isolated from *Metaxya rostrata* Induces Active Cell Death in Colorectal Tumor Cells

**DOI:** 10.1371/journal.pone.0065745

**Published:** 2013-06-11

**Authors:** Kerstin P. Kainz, Liselotte Krenn, Zeynep Erdem, Hanspeter Kaehlig, Martin Zehl, Wilfried Bursch, Walter Berger, Brigitte Marian

**Affiliations:** 1 Medical University Vienna, Department of Medicine 1, Institute of Cancer Research, Vienna, Austria; 2 University of Vienna, Department of Pharmacognosy, Vienna, Austria; 3 University of Vienna, Department of Organic Chemistry, Vienna, Austria; Sapporo Medical University, Japan

## Abstract

*Metaxya rostrata* C. Presl (Metaxyaceae) is a common tree fern in Central and South America that is used for the treatment of intestinal ulcers and tumours in ethnic medicine. Using a bioactivity-guided strategy 2-deprenyl-rheediaxanthone B (XB) has been isolated as one of the active principles in this plant. XB induced loss of cell viability in colorectal cancer cell lines at IC_50_ concentrations of 11–23 µM. This was caused by both accumulation of cells in the G2- and S-phase as well as by induction of active cell death in a time and concentration-dependent manner. Cells exposed to XB were incapable of undergoing regular mitosis due to down-regulation of FoxM1 and absence of chromosome condensation. The apoptosis-related proteins Bcl_2_ and Bcl_xl_ were up-regulated so that Caspase 3 was not activated and classical apoptosis was not observed. However, XB triggered damage pathways down-stream of ATR and activated Caspase 2 causing cell death by a mechanism similar to mitotic catastrophe. Our observations are the first to show the cytotoxic activity of 2-deprenyl-rheediaxanthone B and indicate that XB is an interesting new lead compound for cancer therapy that merits further development.

## Introduction

There has been considerable progress in cancer therapies and prognosis has been improved for several malignancies - e.g. leukemias, breast cancer – while for other tumours - e.g. melanomas, late stage colon cancer - innovative therapeutic options are still urgently needed [1]. Consequently, cancer therapy is one of the most addressed issues in drug discovery. Over the past decade efforts went in two directions – targeted therapeutics and optimized combination chemotherapy. Natural products are an important source of anticancer agents, providing over 50% of the drugs in present clinical use [2] and many of these come from tropical plants used in ethnic medicine [3].

Colorectal cancer is among the most common malignant diseases in the western world and therapy is still difficult at advanced stages. Today the standard therapy consists of 5-fluorouracil in combination with levamisol, oxaliplatin or irinotecan [4], [5]. In addition antibodies targeting the epidermal growth factor receptor and the vascular endothelial growth factor have been introduced and improved prognosis for colon cancer patients [6]. In spite of this progress therapy of advanced colorectal cancers is still not satisfactory due to inherent mechanisms of therapy resistance in colorectal tumour cells - such as mutation of p53 [7] and over-expression of Bcl_2_ [8], [9], as well as multidrug-resistance genes [10], [11].

Therefore we report the bioactivity-guided isolation and biological characterization of a rare bioactive natural compound from the tree fern *Metaxya rostrata* C. Presl (Metaxyacea) that is used in traditional Costa Rican medicine for the treatment of intestinal diseases such as ulcers and tumours [12].

## Materials and Methods

### Extraction and Isolation

Rootlets of *Metaxya rostrata* C. Presl were collected at the research center of the University of Vienna in La Gamba in south-western Costa Rica, and authenticated in the Herbarium of the Museo National in San Jose by Dr. W. Huber (University of Vienna). Voucher specimens (Voucher number: MR0203) are deposited at the Herbarium of the Department of Pharmacognosy, University of Vienna, Austria.

920 g dried rootlets of *Metaxya rostrata* were pulverized and extracted by sonification with CH_2_Cl_2_ yielding 5.3 g CH_2_Cl_2_-extract. The extract was subjected to column chromatography on Sephadex LH-20 under elution with EtOAc (column diameter and height: 4 cm and 75 cm) to obtain 16 subfractions CC-1 to CC-16. In this bioactivity guided approach, after every step of fractionation the obtained fractions were evaluated for their effect on cancer cell viability on SW480. The most active fractions were chosen for further separation. Fraction CC-14 showed highest activity reducing the cell viability in a significant manner at concentrations of 6 µg/ml. Fraction CC-14 was further fractionated using solid phase extraction. It was applied on the SPE-cartridges (C-18, 20 ml, Bond Elut, Varian) and elution was performed sequentially with 4 reservoir volumes each of 60%, 70%, 80%, 90% and 100% MeOH. The fractions of identical polarity were pooled and evaluated for their effect on cancer cell viability. The 70% MeOH-fraction (CC-14/70) was the most active one decreasing cell viability significantly from a concentration of 0.6 µg/ml upwards. The major compound in HPLC analysis at Rt 24.5 min was isolated and identified as 2-deprenyl-rheediaxanthone B using MS and NMR analysis (**Figures S1-S8, Table S1 in Text S1**). As 2-deprenyl-rheediaxanthone B was the major compound with a purity of more than 98% in this fraction, it is certain that the high activity in the bioassay is due to that rare natural compound.

### Chemical Analysis

HPLC was performed on a Shimadzu instrument using LC solution software.

Column: LiChroCART Lichrospher 100 250×4 mm, RP-18e, 5 µm (VWR, Vienna, Austria), eluent A: 1% formic acid in water and eluent B: acetonitrile; flow rate: 1 ml/min; gradient program: 40% B (0 min), 40% B (15 min), 60% B (25 min), and 60% B (30 min); detection modes: UV and E-LSD.

Analytical-grade solvents for extraction and fractionation were obtained from VWR (Vienna, Austria). NMR and MS details are given in supplementary material.

### Cell Lines

SW480, SW620, DLD1, HCT116, and Caco2 colon carcinoma cells were obtained from ATCC and were kept under standard tissue culture conditions using minimal essential medium (MEM) containing 10% fetal calf serum (FCS). F331 cells are fibroblasts isolated from fetal colon [13] and were kept under standard culture conditions using Dulbeccos minimal essential medium (DMEM) supplemented with 10% FCS. LT97 cells were established in our group from early adenoma tissue and grown in a semi-defined medium [14].

### Determination of Viability and Cytotoxic Effect

Cells were plated at 5×10^4^/well in 24-well plates, left to attach for 24 h (SW480, SW620, CaCo2, DLD1, HCT116) or 72 h (F331, LT97). When cultures were semi-confluent they were exposed to XB for 48 hours (stock solutions were prepared in DMSO and stored at –20°C). Control media contained the appropriate volume of DMSO. Cell viability was determined by MTT or neutral red uptake with both methods yielding similar results [15].

### Hoechst Staining

After removal of the neutral red dye, fixed cells were stained with 800 ng/ml Hoechst 33285. Nuclei with chromatin condensed at the nuclear margin or in the centre of the nucleus were classified as apoptotic [15].

### Cell Cycle Analysis

Cells were trypsinized and homogenized with 0.5 M citric acid and 0.5% Tween-20 and the nuclei isolated by centrifugation. After staining with 500 µg/ml PI (Sigma) containing 100 µg/ml RNAse, nuclear DNA content was analyzed by flow cytometry on a FACSCalibur (Becton Dickinson, Mountain View, CA) equipped with a 15 mW argon laser exciting at 488 nm. CellQUEST software was used for data acquisition and MOD-FIT (Becton Dickinson) for data evaluation [15].

### Determination of Mitochondrial Membrane Potential (MMP)

MMP was measured by the lipophilic cationic probe JC-1 (Alexis, Lausanne, Switzerland) that is selectively concentrated in intact mitochondria to form multimer J-aggregates emitting fluorescent light at 590 nm. The monomeric form emits light at 527 nm after excitation at 490 nm. Cells were incubated in MEM with 10% FCS containing 10 µg/ml JC-1 for 10 min at 37°C in the dark. Stained cells were washed, resuspended in PBS and immediately analyzed by flow cytometry [15].

### Western Blot

Cells were washed twice with ice-cold PBS +10 mM NaF and 500 µM o-Vanadate and homogenized in lysis buffer [50 mM Tris/HCl (pH 7.4), 500 mM NaCl, 1% NP-40, 0.5% Na-DOC, 0.1% SDS, 0.05% NaN3, 10 mM NaF, 500 µM o-Vanadate] supplemented with 20 µg/ml complete protease inhibitor cocktail (Roche). Aliquots containing 50 µg of protein were analyzed by electrophoresis on 12% polyacrylamide gels and transferred to polyvinylidine difluoride membranes.

First antibodies recognised Bcl_2_ (BD biosciences), Caspase 2 (Enzo Life Science, Farmingdale, NY), Bcl_xl_, Cyclin A and E (Santa Cruz Biotechnology), Cyclin B, Chk1, phospho-Chk1, Cdc2, phospho-Cdc2, cleaved PARP (Cell Signaling, Beverly, MA), β-Actin (Sigma, St. Louis, MO), PARP, Cdc25C, FoxM1, phospho-Histone H3, and Caspase 3 (R&D Systems, Boston, MA). Secondary antibody was diluted 1∶5000 (antimouse horseradish peroxidase–linked; Amersham, Aylesbury, UK) or 1∶10000 (antirabbit horseradish peroxidase–linked; Amersham) and detected by the enhanced chemiluminiscence Western blotting detection system (Pierce, Rockford, IL) used for detection of proteins. Chemolumiscence was detected by exposure of Kodak medical X-ray films (Kodak, Rochester, NJ) and developing on an Optimax developing machine (Protec, Oberstenfeld, Germany).

### Statistical Analysis

IC_50_ concentrations were determined by non-linear regression and differential sensitivity by 2-way ANOVA using GraphPad Prism software. All other results were analysed by either ANOVA or Kruskal-Wallis Test as appropriate.

## Results

### Isolation and Chemical Structure

**Figure 1:**
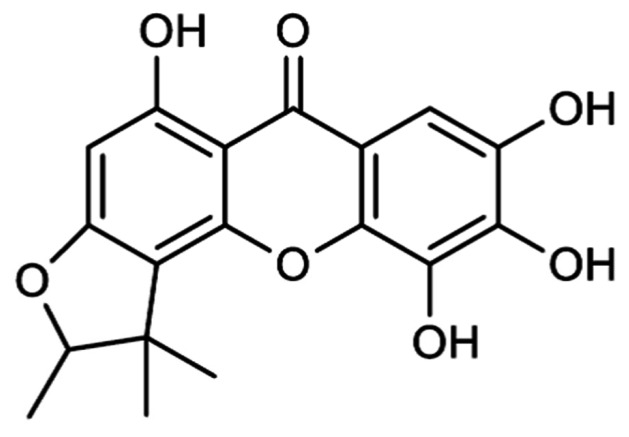
Chemical structure of 2-deprenyl-rheediaxanthone B (XB).

Rootlets of *Metaxya rostrata* were extracted with CH_2_Cl_2_ and fractionated in a bioactivity-guided approach, which focused on the cytotoxicity on SW480 colon carcinoma cells. An active principle was isolated by different chromatographic methods and identified by extensive MS and NMR analysis (find a detailed description of the methods in **Text S1**) as the rare natural compound 2-deprenyl-rheediaxanthone B (XB) (**Figure 1**). Details of the isolation and identification process are given in supplementary materials (**Figures S1–S8, Table S1 in Text S1**).

### Cytotoxic Activity of XB

**Figure 2:**
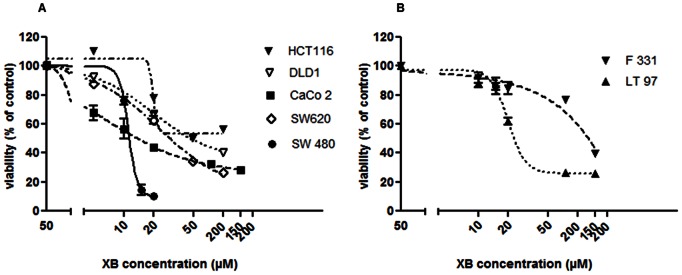
XB-induced cell loss. Semiconfluent cell cultures of HCT116, DLD1, CaCo2, SW620, SW480 (**A**) and F331 and LT97 (**B**) were exposed to increasing concentrations of XB diluted into serum-free treatment medium. Viability was determined 48 hours later by MMT assay. The results shown are the mean±SD pooled from three independent experiments performed in triplicates. *, ** and *** indicate a significant difference as compared to control at p≤0.05, 0.01 and 0.001, respectively.

For biological testing XB was dissolved in DMSO and then diluted directly into the medium of semi-confluent cell cultures. Cytotoxic activity of XB was determined using MTT assays in the colon cancer cell lines SW480, SW620, DLD1, HCT116, and CaCo2 as well as the non-malignant LT97 adenoma cell line and the normal colonic fibroblasts F331. XB significantly reduced cell viability in a concentration-dependent manner in all carcinoma cell lines (**Figure 2**). For 3 of the cell lines – namely SW480, HCT116 and LT97 cells - the dose-response curve was steep and the 95% confidence interval for the IC_50_ concentrations were narrow (**Table 1**). For Caco2, DLD1 and SW620 cell loss was much more gradual with sensitivity becoming less in the sequence Caco2> SW620> DLD1 indicating two different cellular mechanisms of action.

**Table 1:** Sensitivity of CRC cell lines to XB.

cell line	IC_50_ (µM)	95% CI (µM)
SW480	11,10	10.29–11.98
CaCo2	11,02	9.315–13.04
DLD1	20,06	16.66–24.16
SW620	22,98	17.87–29.56
HCT116	19,68	13.36–28.98
LT97	20,18	19.02–21.41
F331	135,9	(Very wide)*

* Very wide indicates that the growth response curve declined too gradual for the graph pad prism software to calculate a 95% CI.

**Figure 3:**
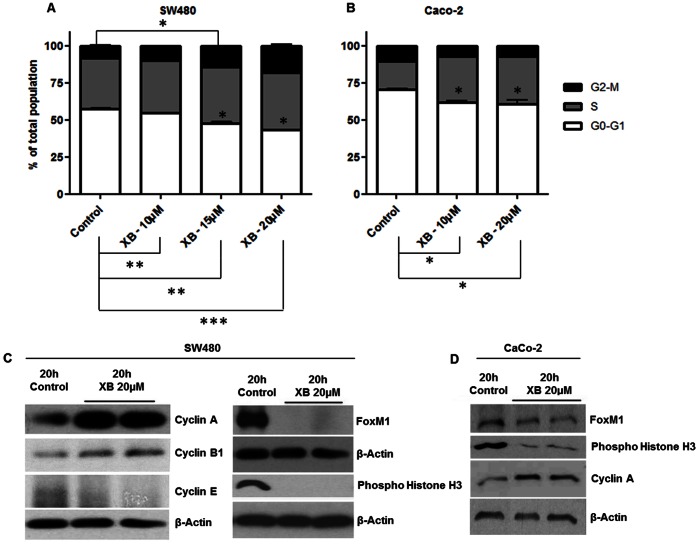
XB-induced cell-cycle blockade. Semi-confluent cultures of SW480 (**A**) and Caco2 (**B**) cells were exposed to the indicated concentrations of XB. 48 hours later nuclei were isolated for the analysis of cell cycle distribution by FACS analysis. The results shown are the mean±SD pooled from three independent experiments. Protein lysates were harvested 20 hours after exposure of SW480 (**C**) and Caco2 (**D**) cells to 20 µM XB and levels of Cyclins A, B1, and E as well as of FoxM1 and phospho-Histone H3 were analysed by western blotting. The figure shows representative examples of two independent experiments.

SW480 and Caco2 were used as typical examples for the investigation of the cellular mechanism underlying the cytotoxic effect of XB. Cell cycle distribution was assessed by FACS analysis and demonstrated a concentration-dependent decrease of the proportion of cells in the G0–G1 phase in both cell lines. In SW480 cultures the percentage of cells in both S-phase and G2-M increased dose-dependently and significantly after 48 h (**Figure 3A**) suggesting an XB-induced G2-M phase cell cycle arrest. In Caco2 cultures cells accumulated in S-phase (**Figure 3B**). In order to confirm the cell cycle blockade, cell cycle regulatory proteins were analysed by western blot from cell lysates obtained after exposure to XB. In SW480 cells the results showed increased levels of Cyclin A and Cyclin B1, while Cyclin E was decreased compared to the control (**Figure 3C**) confirming the G2-M phase cell cycle arrest seen with FACS analysis. The increase of Cyclin A could be confirmed in Caco2 cells (**Figure 3D**).

Protein levels of the forkhead transcription factor FoxM1 - an important regulator of gene expression during the G2 phase and mitosis – was highly expressed in SW480. The protein was no longer detectable in XB exposed SW480 cells, however. In addition, phosphorylation at Ser10 of Histone H3 which is tightly correlated with chromosome condensation was blocked by XB (**Figure 3C**). In Caco2 control cells the FoxM1 level was lower than in SW480 and the impact of XB on both FoxM1 and histone phosphorylation was less pronounced (**Figure 3D**).

**Figure 4:**
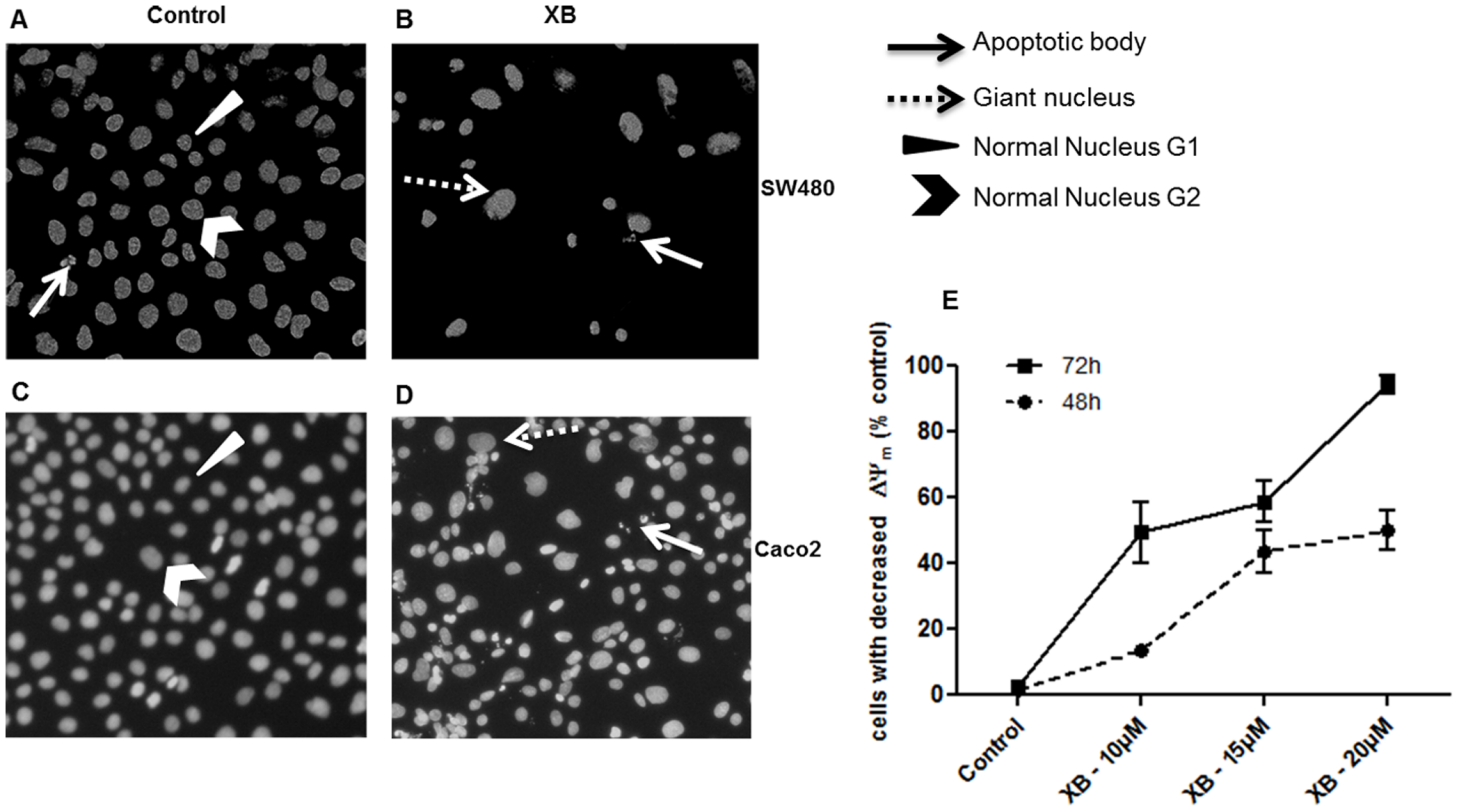
XB-induced active cell death. **A, B, C, D**: In a parallel experiment cultures were fixed after 48 hours of XB-exposure and stained with Hoechst 33258 for visualisation of nuclear morphology (**A**: control SW480, **B**: XB treated SW480, **C**: control CaCo2, **D**: XB treated CaCo2). **E**: Semi-confluent cultures of SW480 were exposed to the indicated concentrations of XB and harvested 48 and 72 hours later for determination of mitochondrial membrane potential (MMP). The results shown are the mean±SD pooled from three independent experiments.

Staining of cells with Hoechst 33258 revealed the appearance of few pycnotic and fragmented nuclei in XB-exposed SW480 and Caco2 cells. However, the most prominent change was an increase in the size of the nuclei compared to the control (**Figure 4A–D**).

Involvement of the mitochondrial pathway of apoptosis induction in XB-induced cell loss was investigated by JC-1 staining and FACS analysis. SW480 cells treated with increasing concentrations of XB for 48 and 72 h showed a dose- and time-dependent increase in cells that have lost MMP compared to the control (**Figure 4E**). After 48 h the fraction of these cells was about 18% with 10 µM of the compound and about 50% at concentrations of 15 and 20 µM. At 72 h 10 and 15 µM XB were sufficient to produce 50% mitochondrial membrane depolarisation and almost all cells had depolarised at a concentration of 20 µM. The IC_50_ concentration for loss of MMP was calculated to be 11.7 µM (CI95% 10.1–13.5) at the 48 h time point.

**Figure 5:**
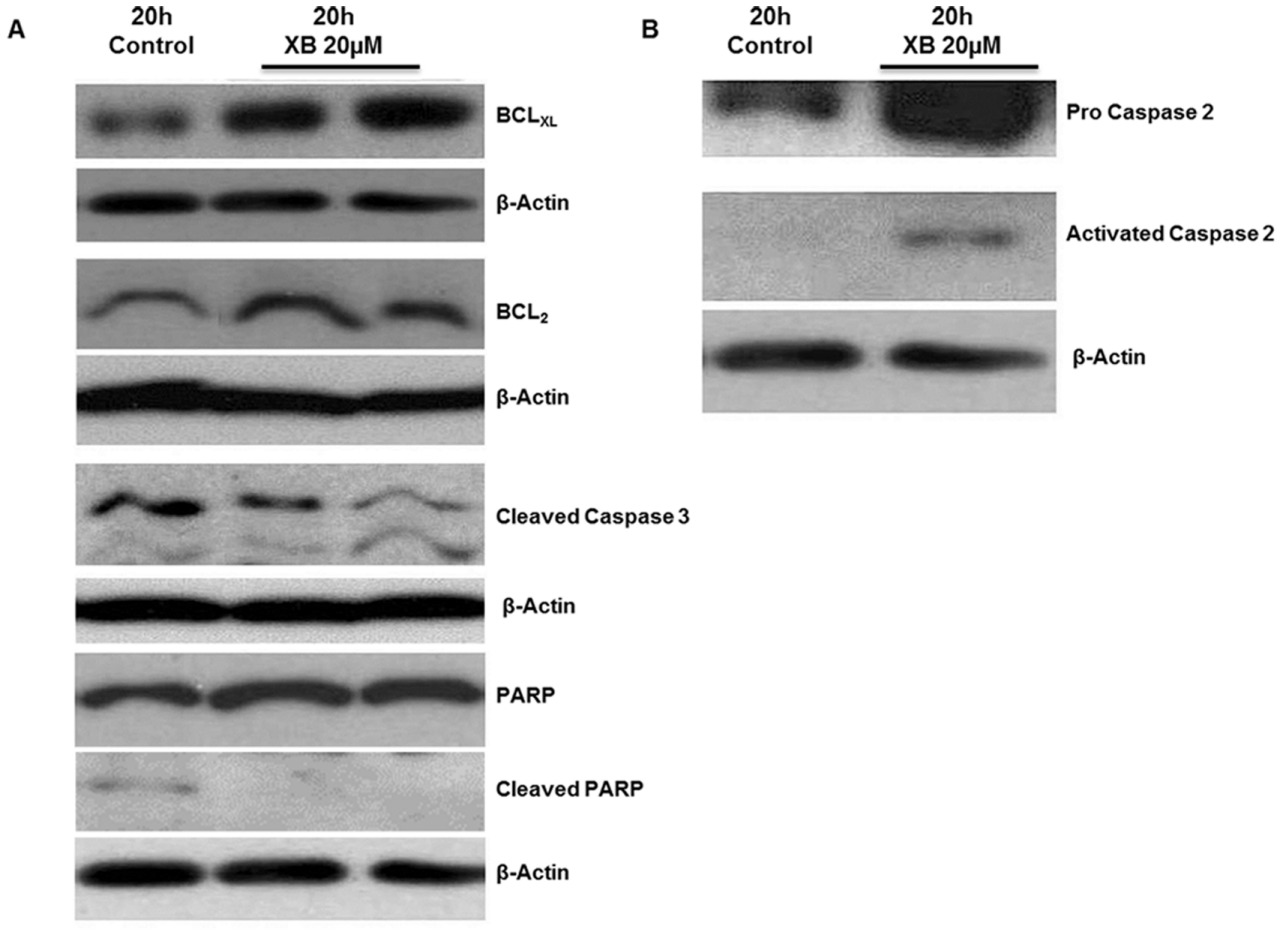
XB does not induce classical apoptosis. Protein lysates were harvested 20 hours after exposure to 20 µM XB and levels of Bcl_xl_, Bcl_2_, activated Caspase 3, PARP and cleaved PARP (**A**) as well as pro Caspase 2 and activated Caspase 2 (**B**) were analysed by western blotting. The figure shows representative examples of two independent experiments.

Analysis of apoptosis-related proteins in SW480 cells by western blot demonstrated slight increases in the levels of the pro-apoptotic proteins BAX and BAK as well as a slight decrease of Survivin as compared to the control (data not shown). The anti-apoptotic proteins Bcl_xl_ and Bcl_2_ were up-regulated and activated Caspase 3 was slightly reduced compared to the control. In agreement with this observation XB did not induce but slightly diminished cleavage of Poly(ADP-ribose) polymerase (PARP) (**Figure 5A**). By contrast, an increase was observed in the levels of both Pro-Caspase 2 and activated Caspase 2 (**Figure 5B**).

**Figure 6:**
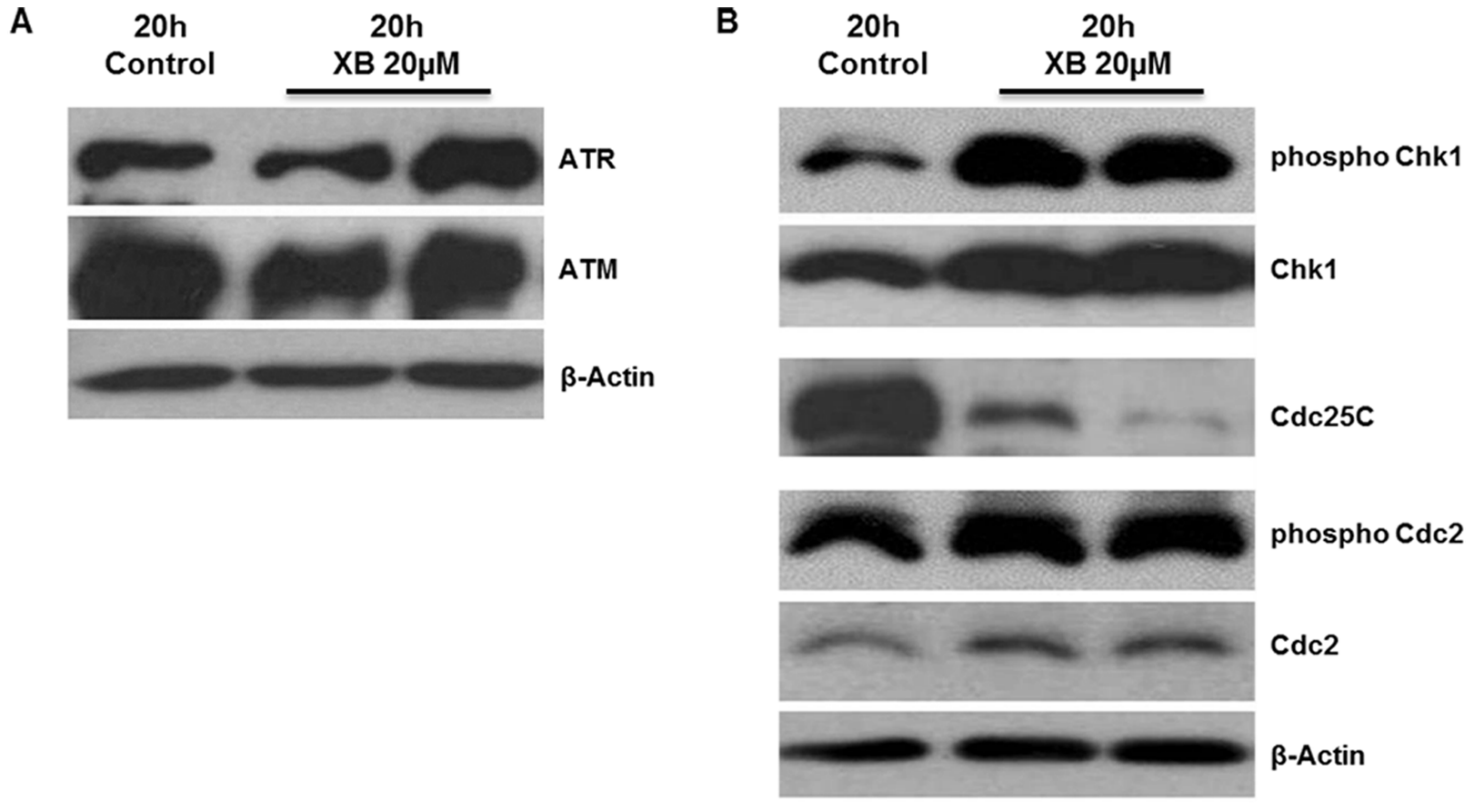
XB effects damage checkpoint proteins. Protein lysates were harvested 20 hours after exposure to 20 µM XB and protein levels of ATR and ATM (**A**) as well as protein levels of Cdc25C and phosphorylation of Chk1 and Cdc2 (**B**) were analysed by western blot. The figure shows representative examples of two independent experiments.

Induction of XB-induced damage response was investigated by determination of the DNA damage markers by phospho-specific western blot analysis. The results showed a slight increase of ATR but not ATM protein level (**Figure 6A**). Down-stream of ATR an increase in both total and phosphorylated Chk1 and a slight increase in total and phosphorylated Cdc2 was observed. Cdc25C was clearly decreased compared to the control (**Figure 6B**).

## Discussion

2-deprenyl-rheediaxanthone B (XB) is a rare natural compound and has been isolated before only from two species of the family of the *Guttiferae*, from the roots of *Rheedia benthamiana* Pl. Triana [16] and the roots of *Hypericum roeperanum* Schimp. ex A. Rich. and also from *Garcinia viellardii* Pierre (*Clusiaceae*) [17]. Hostettmann and his group showed an anti-fungal activity against *Candida albicans* for this compound [18]. In addition, XB has been tested for antimalarial activity [17] and effects on angiogenesis [19]. Further biological activities of 2-deprenyl-rheediaxanthone B have not yet been studied.

In our study the compound was isolated from the Costa Rican tree fern *Metaxya rostrata*. The plant is common in the rain forests of Central and South America and used in ethnic medicine for the treatment of intestinal ulcers and tumours [20], which made it a promising source of cytotoxic lead compounds. XB is not the only bioactive compound in *Metaxya rostrata*. The polyphenols cinnamtannin B-1 and aesculitannin B have been isolated in a previous study. With an IC_50_ of about 11 µM XB is much more effective in SW480 cells than the polyphenols, that induced cell death with an IC_50_ of about 50 µM [20]. Viability of other colon carcinoma cell lines was similarly reduced by XB with IC_50_ concentration ranging from 10–23 µM. Non-malignant colon adenoma cells also had an IC_50_ concentration of about 20 µM, while normal human fibroblasts were less sensitive indicating at least some specificity for (pre-)malignant cells.

XB induced a cell cycle block in S and G2/M as characterised by increased levels of Cyclins A and B, while Cyclin E decreased. Protein levels of Cdc2, the kinase partner of Cyclin B, were increased, but Cdc2 phosphorylation was little affected. In addition, Cdc25C that activates CyclinB/Cdc2 in G2 [21] was decreased. Together these observations place the exact point of the cell cycle block at S and G2. This may be due to down-regulation the FoxM1 that was observed in both SW480 and Caco2 cells. The transcription factor is essential for the progress of the cells into mitosis [22] and in both cell lines phosphorylation of Histone 3– a prerequisite of chromosome condensation in mitosis [23] – was much reduced. SW480 and Caco2 cells differ in their control levels of FoxM1 protein, which are much higher in SW480. The XB-induced decrease of FoxM1 level was less in Caco2, however, as was the reduction of Histone phosphorylation. This correlates with both a more gradual dose-response curve with regard to loss of cell viability and a cell cycle block rather in S than in G2. Whether this is a mechanistic consequence of the lesser FoxM1 level warrants further investigation which could not be performed in this study due to the limited amount of XB available.

In addition XB-induced cell death was an active process involving loss of MMP. The IC_50_ concentration for this process was in the same range (10–13 µM) as the IC_50_ for loss of viability indicating that the active cell death component is the main mechanism for cell loss. The underlying cellular mechanism of cell death was not indicative of apoptosis, however. In spite of the time and concentration-dependent loss of MMP, no activation of Caspase 3 occurred and PARP degradation was even reduced. In addition, Bcl_2_ and Bcl_xl_ were not decreased but slightly up-regulated. By contrast the caspase activated by XB was Caspase 2. In addition the compound induced the damage response markers ATR, Chk1 and Cdc2, and down-regulated Cdc25C. Together these alterations are characteristic for the induction of mitotic catastrophe [24], [25]. The accumulation of giant nuclei indicates that the cells go through several cycles of DNA-replication in spite of their inability to initiate chromosome condensation and mitosis.

While no information has previously been available on the effect of XB in other tumour types, different prenylated xanthones have been reported to possess activity against MCF-7, NCI-H460 [26], HL60 leukemia cells [27], HeLa and MDA-MB-231 cancer cell lines [28]. α-Mangostin, the best studied prenylated xanthone from *Garcinia mangostana* L., induced cell-cycle arrest in G1 and apoptosis through the activation of the intrinsic pathway in DLD-1 human colon cancer cells [29]–[31]. In general various xanthone derivatives exert growth inhibitory and cell death inducing effects in different cell lines and the cellular mechanisms involved can vary widely. In addition to classical apoptotic pathways e.g. [32]–[34] caspase-independent forms of cell death [35] and autophagy [36] have been observed.

Pyranoxanthones are suspected to induce apoptosis by directly binding to Bcl-family proteins [37]. Other xanthone derivatives interfered with down-stream survival signalling [29], [34], induced oxidative stress [32], [38] or directly interacted with the DNA [33], [39]. XB triggered the damage response pathway in SW480 cells by activating Chk1 indicating the involvement of oxidative or replicative stress in the cell response. In a p53-defective tumour cell like SW480 this should cause a G2 cell cycle arrest by inactivating cyclin B/cdc2 kinase activity [40], which is in agreement with our results. It should also protect cells from DNA-damage induced cell death [41] and actually we did not observe classical damage-induced apoptosis in our experiments. By contrast the cells form polyploid giant nuclei without any indication of chromosome condensation indicating an additional target at the G2/M border. This target could be FoxM1 that has gained much interest in this context [42] and is down-regulated in our experiments.

Mitotic catastrophe (MC) has gained interest as response to cancer drugs as well as radiation especially in solid tumours where the induction of classical apoptosis turned out to be rare (for review see [43]). In this context it is a considerable advantage that MC can also be induced independent of p53 function. It can be regarded as a delayed response of p53-mutant tumours that are resistant to replicative damage. Compounds inducing MC like XB could improve the therapy options for solid tumours bearing an inactive p53 protein [24], [44] including colon cancer.

A difficulty arises from the rarity of the substance that is mainly contained in the roots of *M. rostrata* and also only at low concentrations. This is the reason why the analysis of the biological mechanisms underlying XB’s cellular mechanism of action had to be limited to proof of principle in this study. It also hampers further development. Subsequent projects have to investigate alternative sources and/or semi-synthetic approaches to produce the compound. Chances are promising, because some several other xanthones have shown growth inhibitory and cell death-inducing effects in different cell lines and trigger various cellular mechanisms of cell death.

## Supporting Information

Figure S1 **LC/MS-analysis of compound XB.** Analysis parameters were: +ESIMS *m/z* 329.1 [M+H]^+^; +ESIMS^2^ (329.1 →) *m/z* 286.9 (21) [M–C_3_H_6_+ H]^+^, 272.9 (100) [M–C_4_H_8_+ H]^+^, 248.9 (3); –ESIMS *m/z* 327.0 [M-H]^–^; –ESIMS^2^ (327.0 →) *m/z* 296.9 [M–CH_2_O–H]^–^ (100). (A) Positive ion mode ESI-MS and (B) MS^2^ (329.1 →) spectrum of compound **XB**.
(TIF)

Figure S2 **LC/MS-analysis of compound XB.** Parameters were as described in Figure S2. Negative ion mode ESI-MS (A) and MS^2^ (327.0 →) spectrum (B) of compound **XB**.
(TIF)

Figure S3 **NMR analysis of compound XB.** ^13^C NMR spectrum (APT) (CD_3_OD, 150.92 MHz).
(TIF)

Figure S4 **NMR analysis of compound XB.** ^1^H NMR spectrum (CD_3_OD, 600.13 MHz).
(TIF)

Figure S5 **(ge)-HSQC spectrum (multiplicity edited) of compound XB (CD_3_OD).**
(TIF)

Figure S6 **(ge)-HMBC spectrum of compound XB (CD_3_OD).**
(TIF)

Figure S7 **(ge)-DQF-COSY spectrum of compound XB (CD_3_OD).**
(TIF)

Figure S8 **NOESY spectrum (800 ms mixing time) of compound XB (CD_3_OD).**
(TIF)

Text S1 **Supplementary information on the isolation and chemical analysis of 2-Deprenyl-rheediaxanthone B (XB).** The text contains the supplemental materials and methods as well as table S1 listing published NMR-data of XB.
(DOC)
