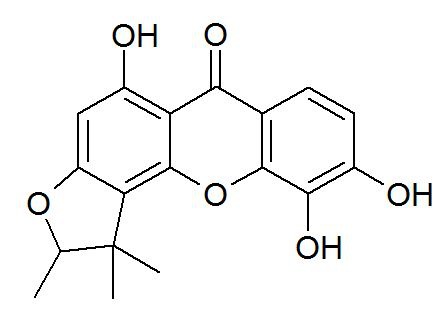# Correction: 2-Deprenyl-Rheediaxanthone B Isolated from *Metaxya rostrata* Induces Active Cell Death in Colorectal Tumor Cells

**DOI:** 10.1371/annotation/f4e4a7b2-f60d-4102-a56f-37666aafd7e1

**Published:** 2013-09-13

**Authors:** Kerstin P. Kainz, Liselotte Krenn, Zeynep Erdem, Hanspeter Kaehlig, Martin Zehl, Wilfried Bursch, Walter Berger, Brigitte Marian

There was an error in Figure 1. The correct version of the figure is available here: 

**Figure:**