# Supplementary figures and images for: 2-Deprenyl-Rheediaxanthone B Isolated from Metaxya rostrata Induces Active Cell Death in Colorectal Tumor Cells
Source: PLoS One. 2013 Jun 11;8(6):e65745. doi: 10.1371/journal.pone.0065745 (PMC3679105; doi:10.1371/journal.pone.0065745)

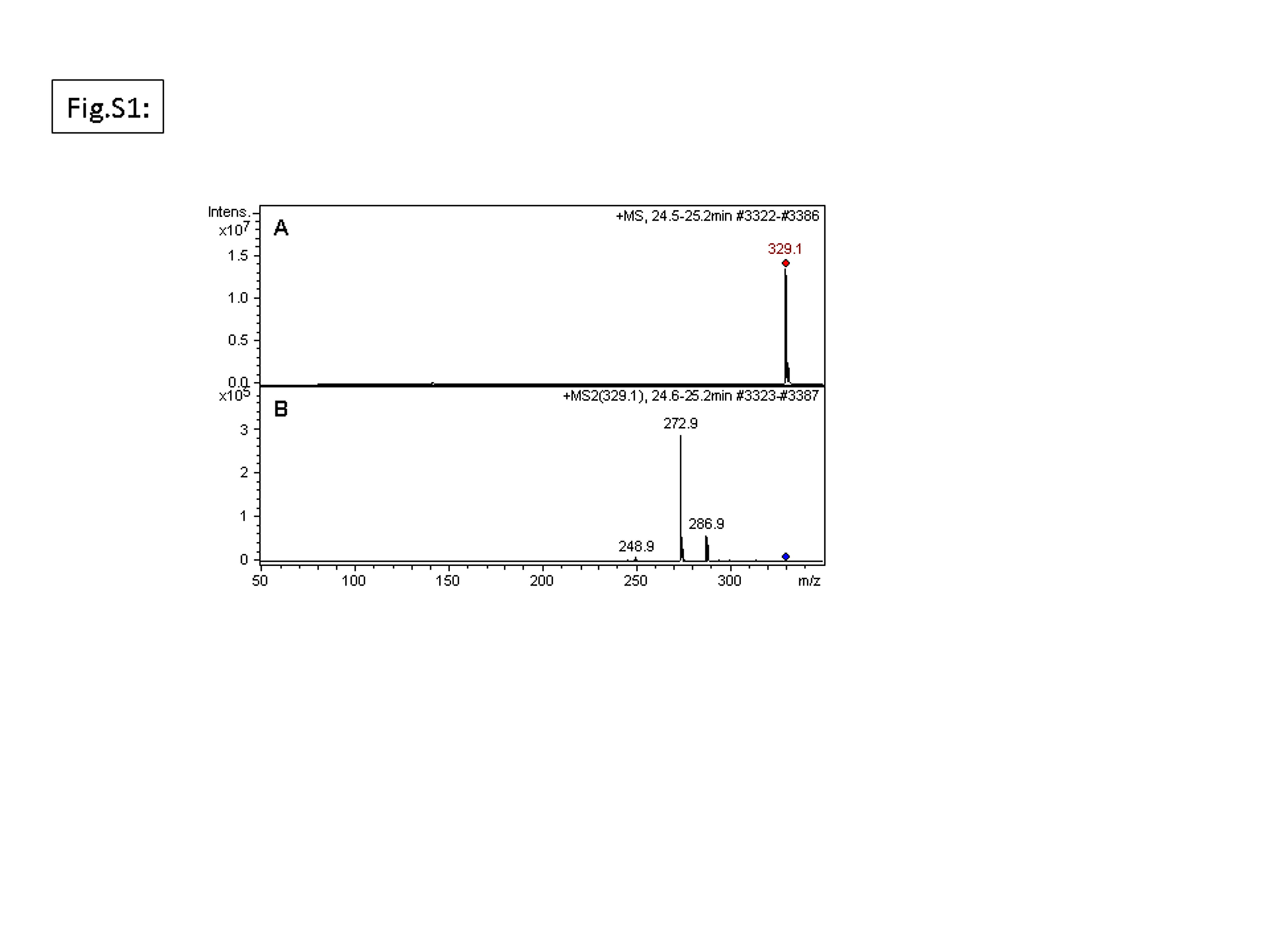

Supplement: Figure S1 — LC/MS-analysis of compound XB. Analysis parameters were: +ESIMS m/z 329.1 [M+H]+; +ESIMS2 (329.1 →) m/z 286.9 (21) [M–C3H6+ H]+, 272.9 (100) [M–C4H8+ H]+, 248.9 (3); –ESIMS m/z 327.0 [M-H]–; –ESIMS2 (327.0 →) m/z 296.9 [M–CH2O–H]– (100). (A) Positive ion mode ESI-MS and (B) MS2 (329.1 →) spectrum of compound XB. (TIF) [file pone.0065745.s001.tif]

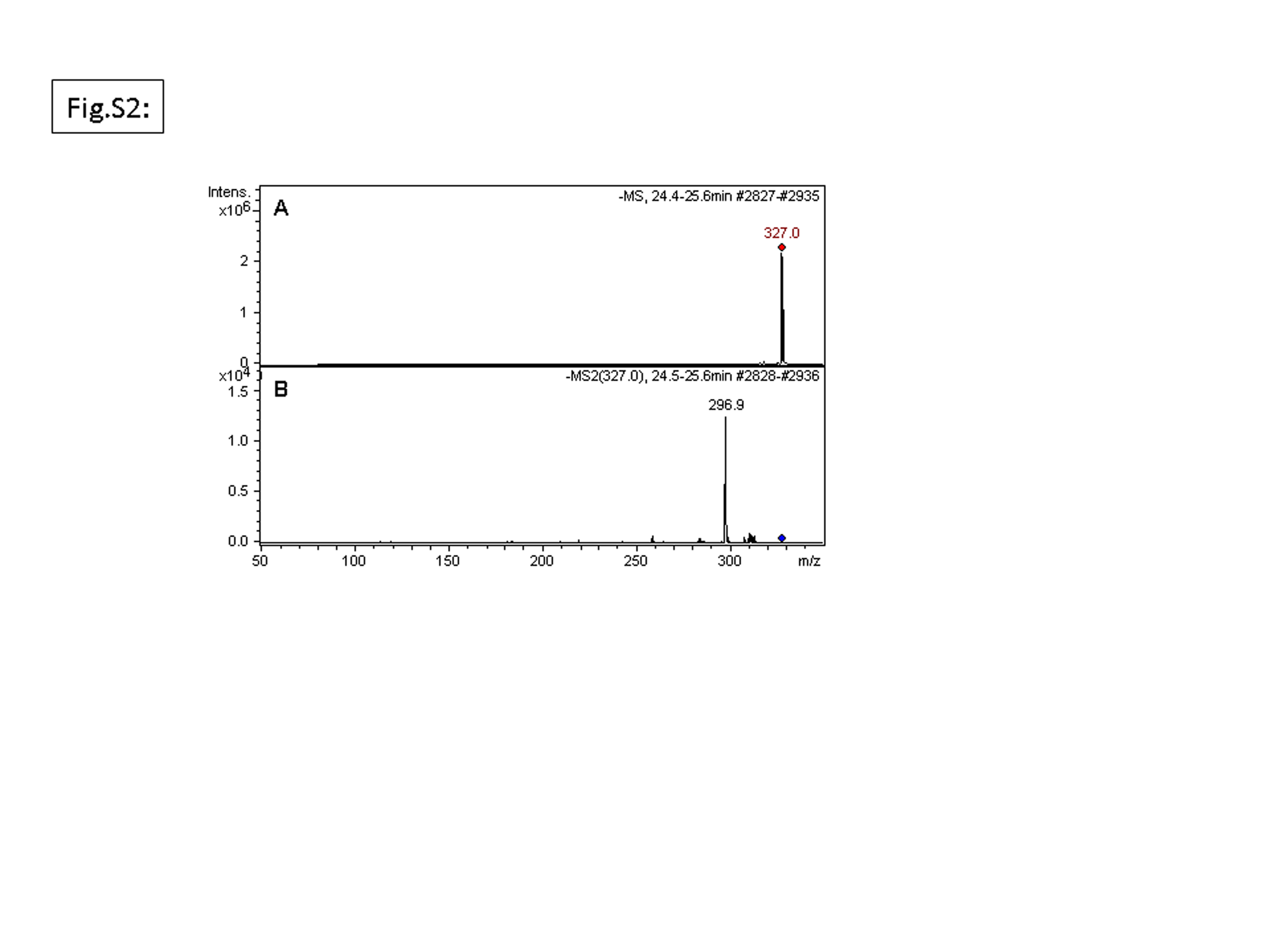

Supplement: Figure S2 — LC/MS-analysis of compound XB. Parameters were as described in Figure S2. Negative ion mode ESI-MS (A) and MS2 (327.0 →) spectrum (B) of compound XB. (TIF) [file pone.0065745.s002.tif]

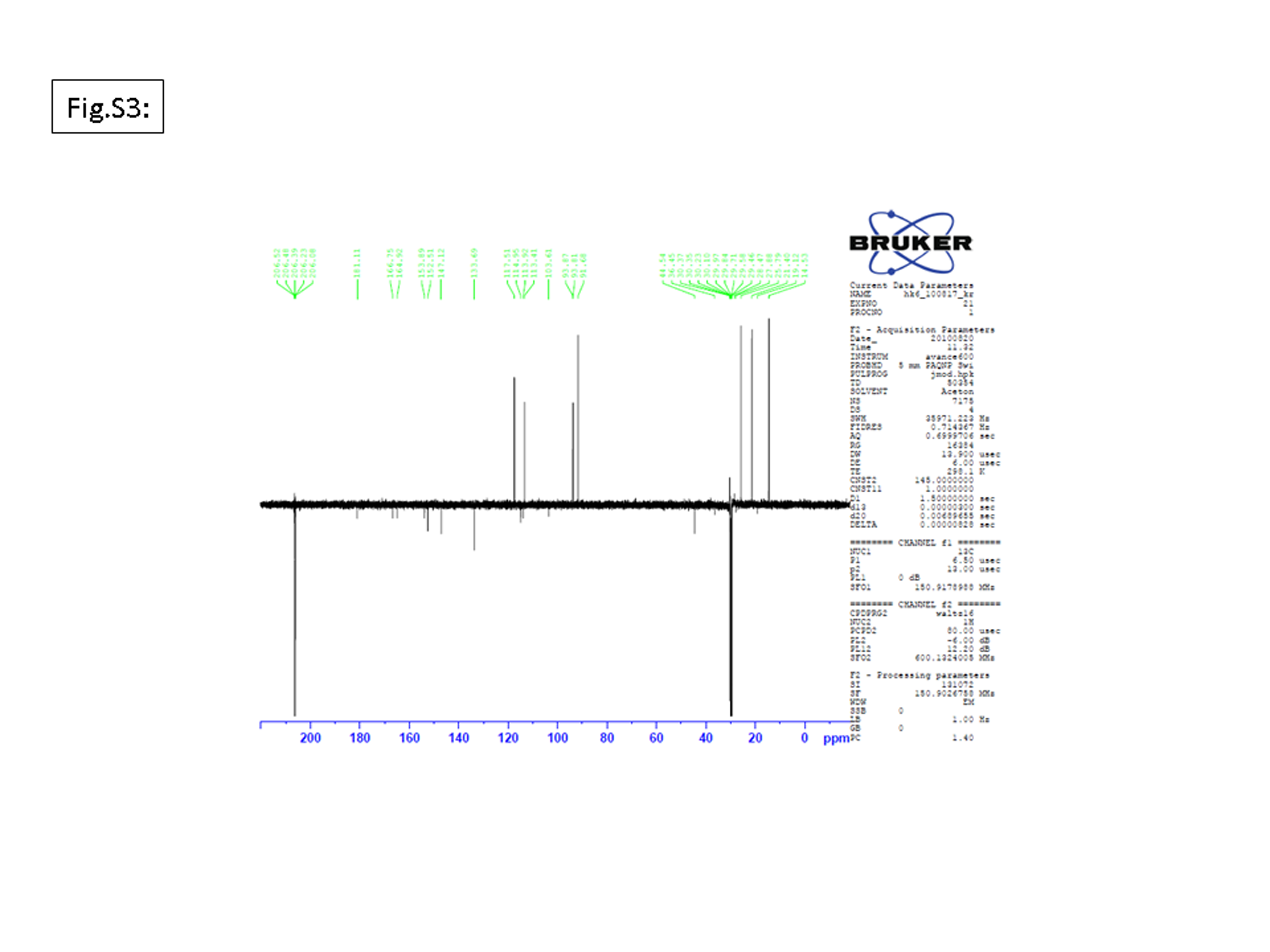

Supplement: Figure S3 — NMR analysis of compound XB. 13C NMR spectrum (APT) (CD3OD, 150.92 MHz). (TIF) [file pone.0065745.s003.tif]

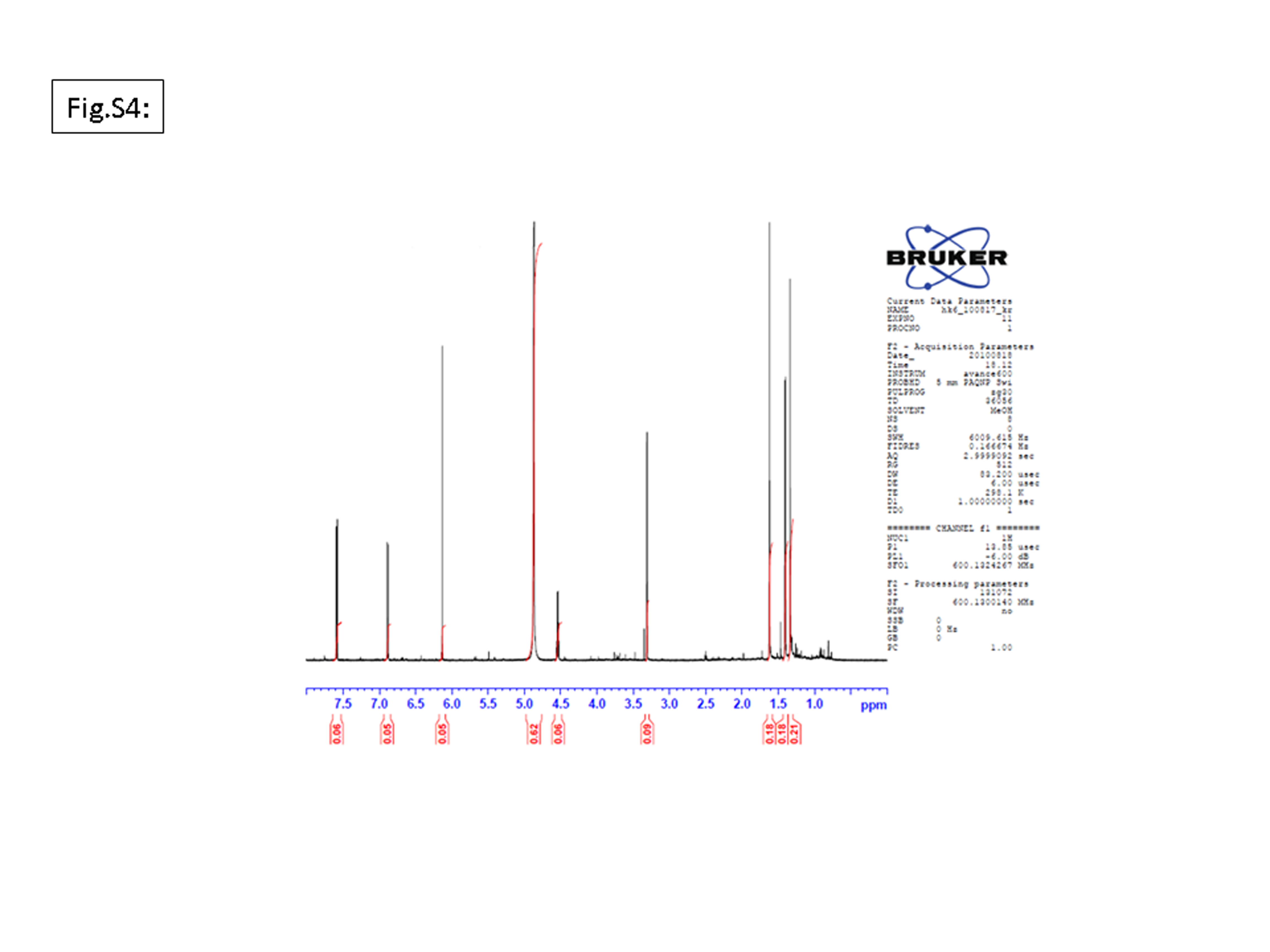

Supplement: Figure S4 — NMR analysis of compound XB. 1H NMR spectrum (CD3OD, 600.13 MHz). (TIF) [file pone.0065745.s004.tif]

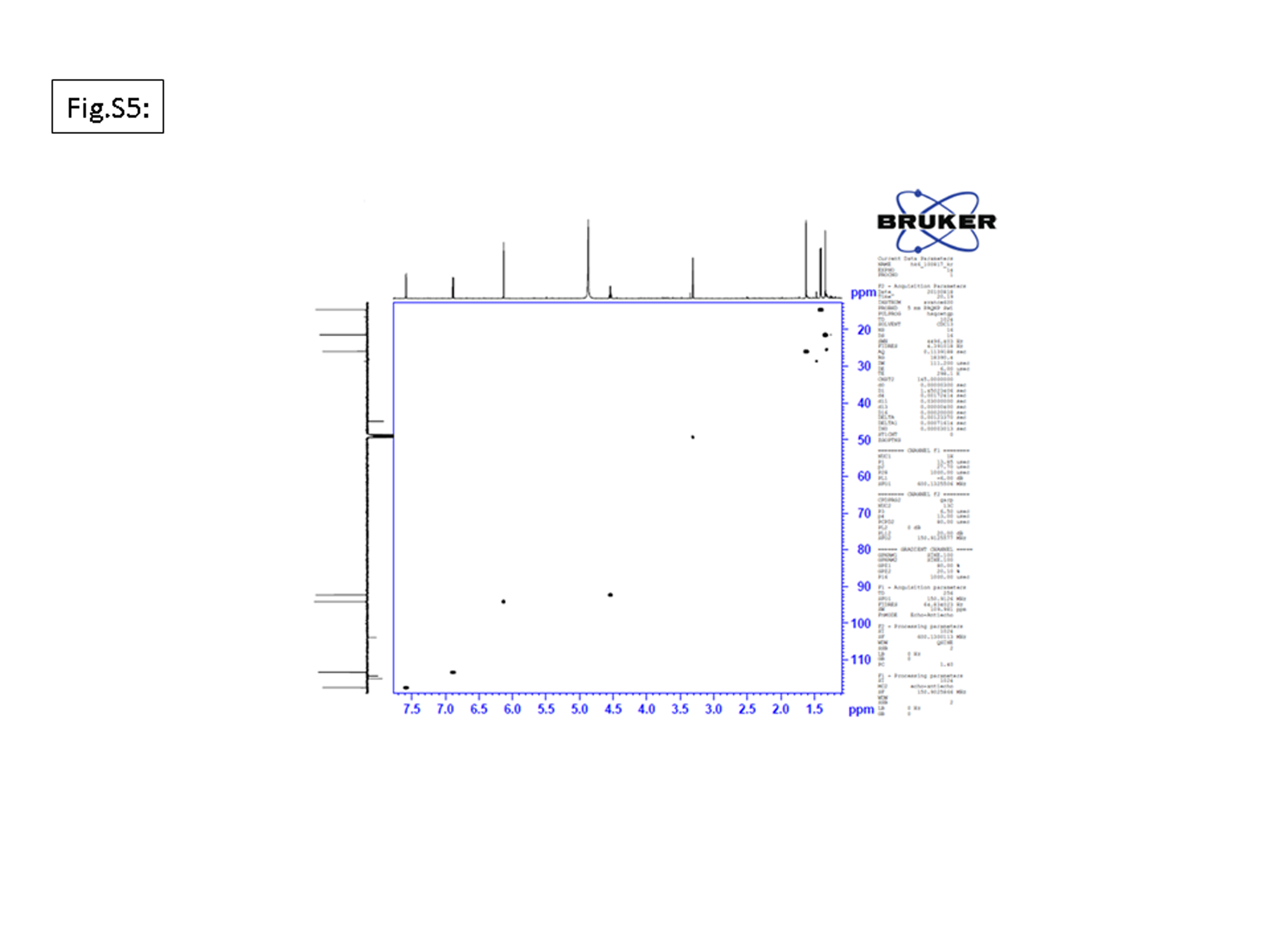

Supplement: Figure S5 — (ge)-HSQC spectrum (multiplicity edited) of compound XB (CD3OD). (TIF) [file pone.0065745.s005.tif]

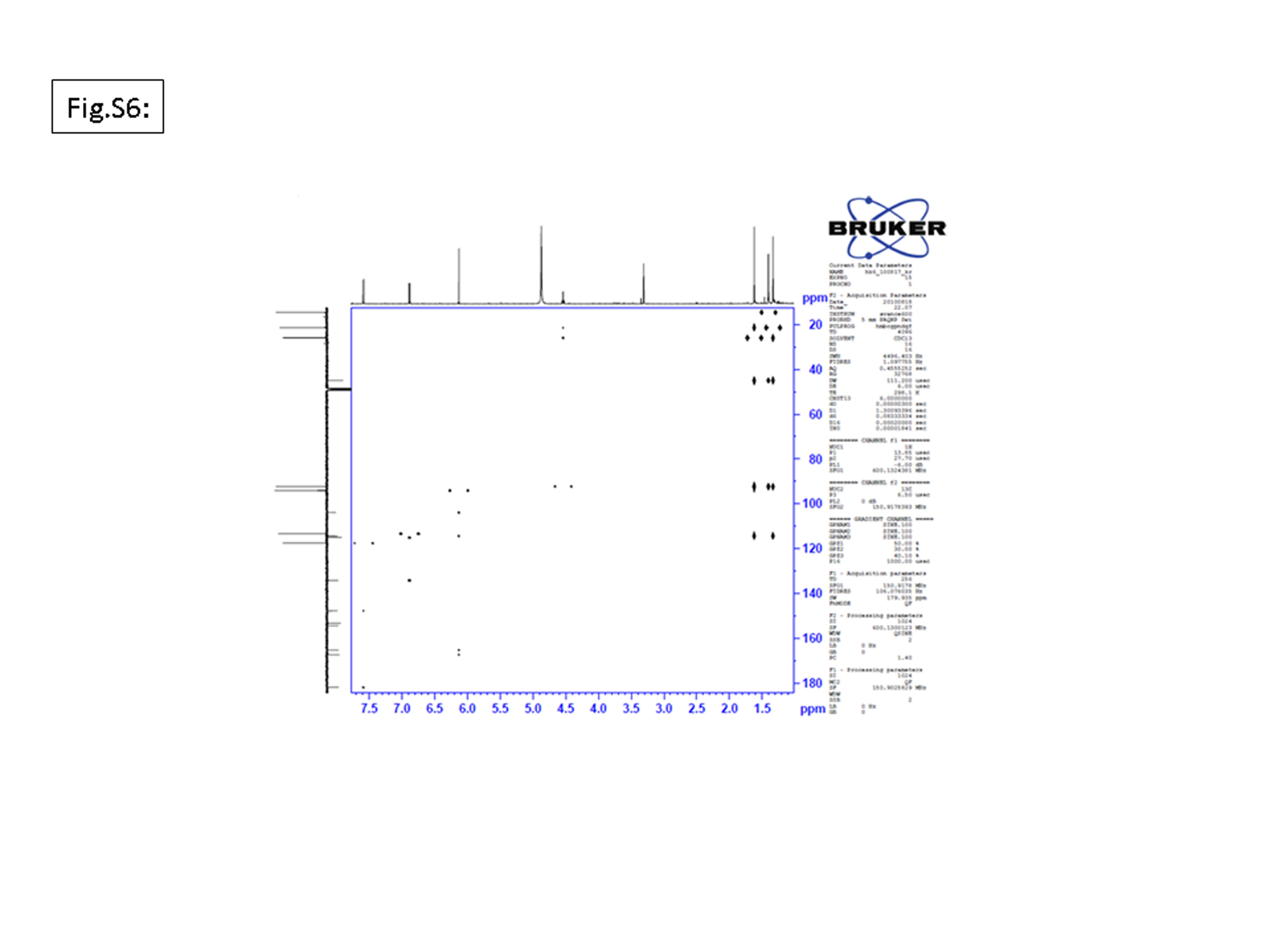

Supplement: Figure S6 — (ge)-HMBC spectrum of compound XB (CD3OD). (TIF) [file pone.0065745.s006.tif]

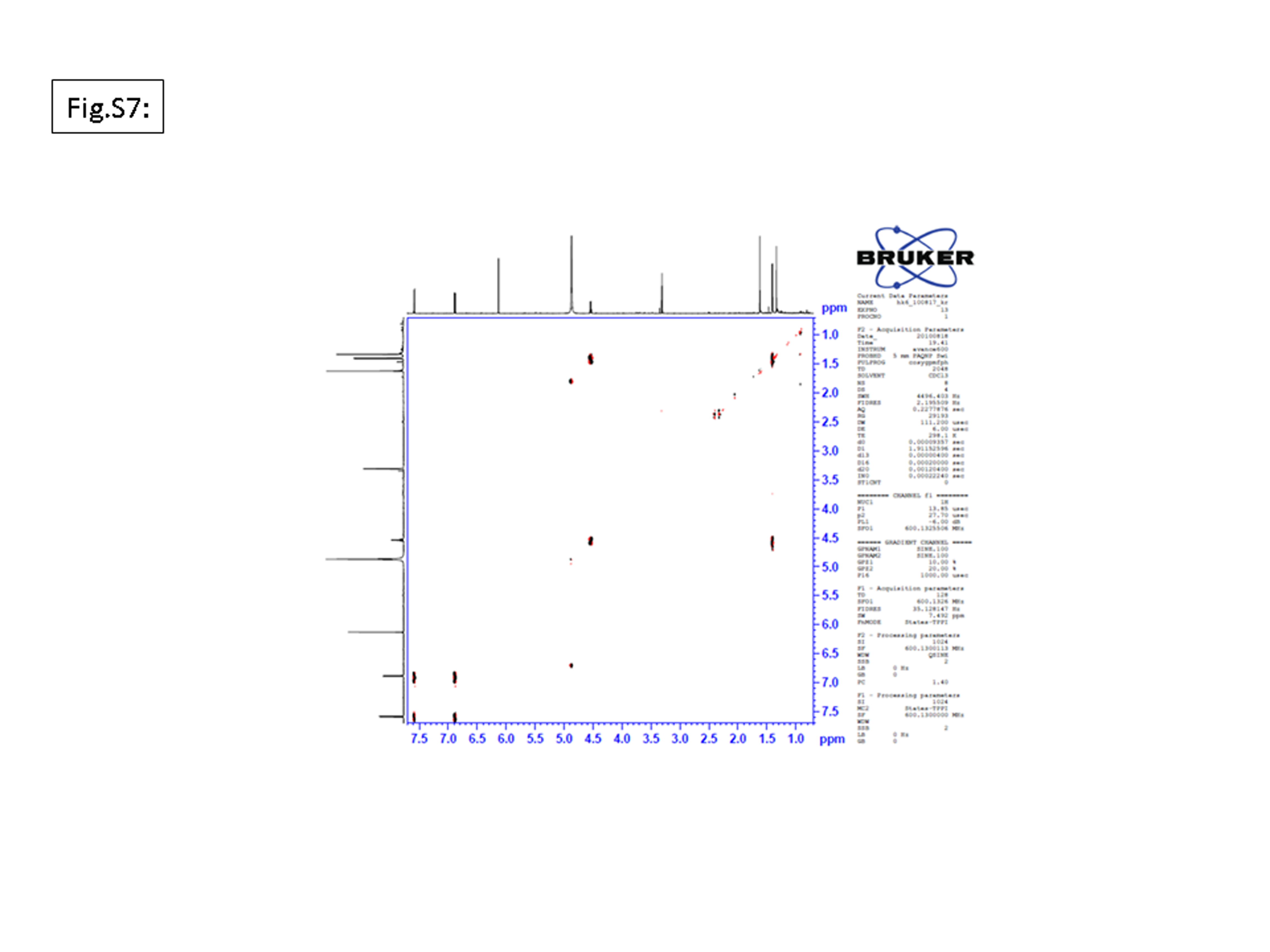

Supplement: Figure S7 — (ge)-DQF-COSY spectrum of compound XB (CD3OD). (TIF) [file pone.0065745.s007.tif]

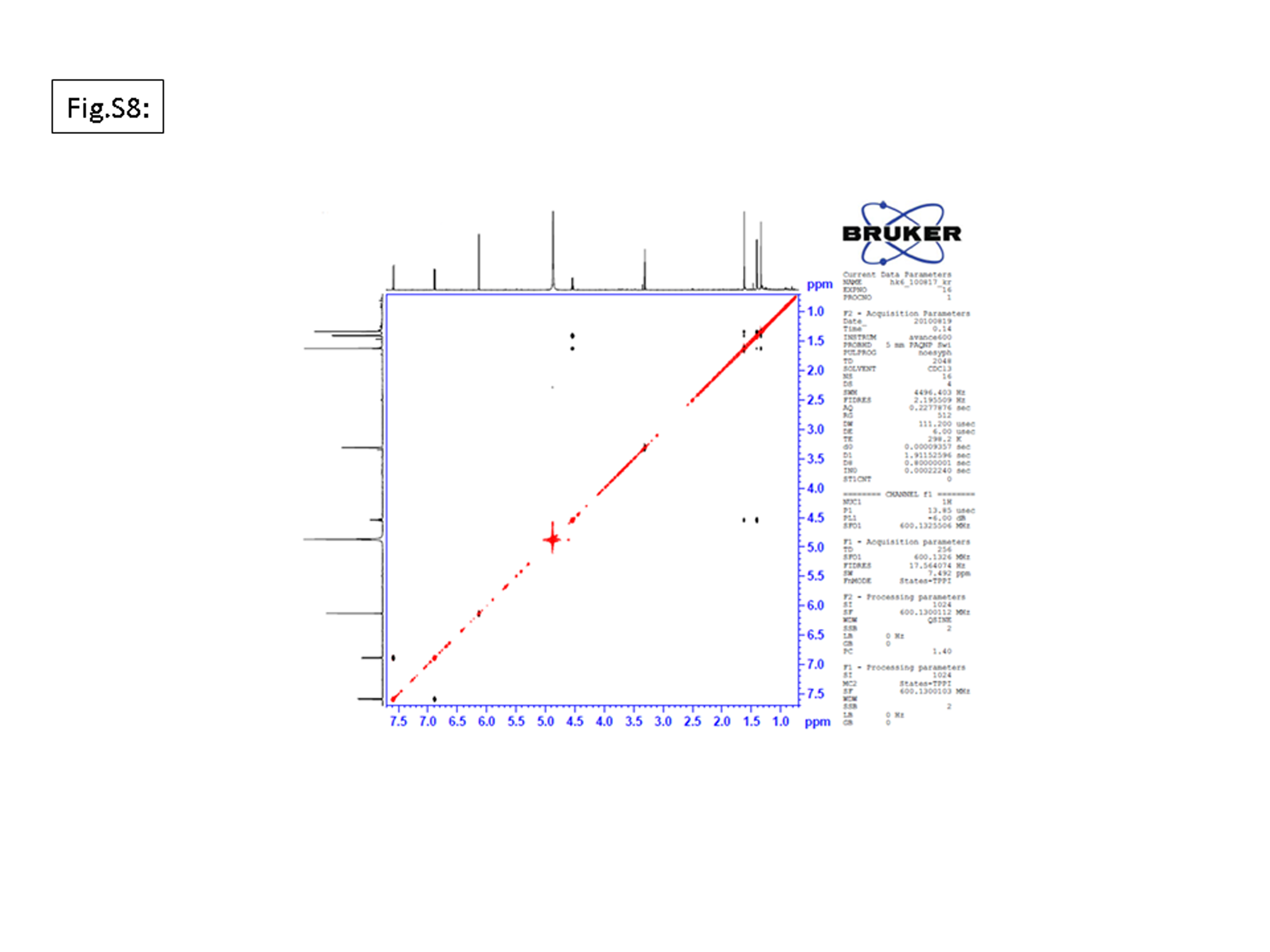

Supplement: Figure S8 — NOESY spectrum (800 ms mixing time) of compound XB (CD3OD). (TIF) [file pone.0065745.s008.tif]
