# Supplementary material for: 2-Deprenyl-Rheediaxanthone B Isolated from Metaxya rostrata Induces Active Cell Death in Colorectal Tumor Cells
Source: PLoS One. 2013 Jun 11;8(6):e65745. doi: 10.1371/journal.pone.0065745 (PMC3679105; doi:10.1371/journal.pone.0065745)
Supplement: Text S1 — Supplementary information on the isolation and chemical analysis of 2-Deprenyl-rheediaxanthone B (XB). The text contains the supplemental materials and methods as well as table S1 listing published NMR-data of XB. (DOC) [file pone.0065745.s009.doc]

**Text S1 - Supplementary Information**

**2-Deprenyl-rheediaxanthone B Isolated from *Metaxya rostrata* Induces Active Cell Death in Colorectal Tumor Cells**

Kerstin P. Kainz^1,2^, Liselotte Krenn^2^, Zeynep Erdem^1^, Hanspeter Kählig^3^, Martin Zehl^2^, Walter Berger^1^, Wilfried Bursch^1^, Brigitte Marian^1^*

^1^ Medical University Vienna, Department of Medicine 1, Institute of Cancer Research, Borschkegasse 8a, 1090 Vienna, Austria

^2^ University of Vienna, Department of Pharmacognosy, Althanstrasse 14, 1090 Vienna, Austria

^3^ University of Vienna, Department of Organic Chemistry, Währingerstrasse 38, 1090 Vienna, Austria

**Correspondence:**

Brigitte Marian

Institute of Cancer Research
Department of Medicine 1
Medical University Vienna
Borschkegasse 8a tel: +43 1 40160 57522
1090 Vienna fax: +43 1 40160 957500
Austria e-mail: [brigitte.marian@meduniwien.ac.at](mailto:brigitte.marian@meduniwien.ac.at)

**Supplemental Materials**

All NMR spectra were recorded on a Bruker Avance DRX 600 NMR spectrometer using a 5mm switchable quadruple probe (QNP, ^1^H, ^13^C, ^19^F, ^31^P) with z axis gradients and automatic tuning and matching accessory. The resonance frequency for ^1^H NMR was 600.13 MHz, for ^13^C NMR 150.92 MHz. All measurements were performed for a solution in fully deuterated methanol or acetone at 298K. Standard 1D and gradient-enhanced (ge) 2D experiments, like double quantum filtered (DQF) COSY, TOCSY, NOESY, HSQC, and HMBC, were used as supplied by the manufacturer. Chemical shifts are referenced internally to the residual, non-deuterated solvent signal for ^1^H (δ = 3.31 ppm for methanol, δ = 2.05 for acetone) or to the carbon signal of the solvent for ^13^C (δ = 49.00 ppm for methanol, δ = 29.84 for the methyl signal of acetone).

LC/MS analyses were performed on an UltiMate 3000 RSLC-series system (Dionex, Germering, Germany) coupled to a 3D quadrupole ion trap mass spectrometer equipped with an orthogonal ESI source (HCT, Bruker Daltonics, Bremen, Germany). HPLC separation was carried out on an LiChroCART® column, 250 × 4.0 mm, LiChrospher® 100 RP-18e, 5 µm (VWR, Vienna, Austria) at 25°C using 1% aqueous formic acid and acetonitrile as mobile phase A and B, respectively. The flow rate was 1.0 mL/min and the following gradient program was used: 40% B (0 min), 40% B (15 min), 60% B (25 min), and 60% B (30 min). The eluent flow was split roughly 1:8 before the ESI ion source, which was operated as follows: capillary voltage: ±3.7 kV, nebulizer: 30 psi (N_2_), dry gas flow: 8 L/min (N_2_), and dry temperature: 340 °C. The mass spectrometer was operated in an automated data-dependent acquisition (DDA) mode to obtain MS^2^ and MS^3^ spectra (collision gas: He, isolation window: 4 Th, fragmentation amplitude: 1.0 V).

UV-VIS spectra were measured on a Beckman DU 640 Spectrophotometer. Infrared spectra were measured on a Perkin Elmer FT-IR 2000 instrument in attenuated total reflection mode using a Golden Gate ATR unit. TLC was performed on silica gel plates (Merck, Germany, 0.25mm, 20x20cm); mobile phases used: (1) EtOAc-formic acid-MeOH-H_2_O (70:8:8:11); (2) CHCl_3_-MeOH-H_2_O (70:22:3); (3) EtOAc-MeOH-H_2_O (81:11:8); detection: (A) anisaldehyde-H_2_SO_4_-reagent; (B) Naturstoff-reagent A/PEG 400 under UV 366.

Table S1: ^1^H-NMR- and ^13^C-NMR data of compound XB (literature data from G. Rath, O. Potterat, S. Mavi, K. Hostettmann: Xanthones from *Hypericum roeperanum*, Phytochem. 1996, 43, 513-520)

^1^H J(H,H) ^13^C ^1^H J(H,H) ^13^C

(MeOH-d_4_) (Acetone-d_6_) Lit.

1 C --- 165.31 --- 164.92 ^a^166.8

2 CH 6.131 s 94.12 6.150 s 93.81 93.8

3 C --- 167.35 --- 166.79 ^a^165.2

4 C --- 114.38 --- 113.92 113.4

4a C --- 154.39 --- 153.89 152.6

4b C --- 147.78 --- 147.12 147.1

5 C --- 134.21 --- 133.69 133.7

6 C --- 153.24 --- 152.51 152.6

7 CH 6.885 d 8.7 113.39 6.990 d 8.7 113.41 113.4

8 CH 7.587 d 8.7 117.58 7.626 d 8.7 117.51 117.5

8a C --- 115.09 --- 114.95 114.9

9 C --- 181.93 --- 181.11 181.1

9a C --- 103.92 --- 103.61 103.5

11 C --- 44.98 --- 44.54 44.5

12 CH_3_ 1.622 s 25.94 1.634 s 25.79 25.8

13 CH_3_ 1.336 s 21.42 1.340 s 21.40 21.4

14 CH 4.540 q 6.6 92.29 4.572 q 6.6 91.68 91.6

15 CH_3_ 1.405 d 6.6 14.57 1.406 d 6.6 14.53 14.5

1 OH --- --- 13.482 s ---
